# Help us make science better with Registered Reports

**DOI:** 10.1038/s41598-020-79467-9

**Published:** 2021-01-21

## Abstract

We are a story telling species. We make sense of the world by looking for order and patterns. So we often see a signal—and build explanatory edifice around it—where there is just noise; and we often see noise where there may well be a signal. This habit of shaping reality into an easy to follow narrative affects even one of the most structured and systematic human activities: science. One of two things tends to happen next. We either ignore the evidence that does not fit our story, or we bend and mould it until it does.

Ignoring the evidence leads to publication bias against negative results. When in 1975 Anthony Greenwald bemoaned the prejudice against the null hypothesis^1^, he recounted the dictionary definition of ‘null’: something of no significance, or consequence. In the minds of many, what did not fit had no value. This struggle against the file drawer effect, as it was named a few years later, was as real when he described it as it is now, nearly five decades later.

Manipulation of evidence can be due to reporting results without transparently revealing all analytical freedoms that we afforded ourselves. This can take many forms, and while we’ve only recently started naming these phenomena, they have been known to scientists for decades, if not centuries. For example, what we would now call *p*-hacking was described by Augustin Cournot in 1843^2^.

Publication bias can be addressed by journals. And so it is: combating bias against negative results, or much-needed replications, is part of the *Scientific Reports* ethos. We don’t judge research based on perceived importance; we don’t make decisions based on priority claims but rather on the robustness of the research; we recognise that research is an incremental process and that it can be unclear which of these increments may help cure disease or lead to the development of interstellar travel.

Questionable research practices are a different story altogether. They can be motivated by unhealthy incentives ingrained into the process of research communication and evaluation^3^. For these incentives to be despatched there must be a promise that assessment and dissemination of research will not depend on the one thing that the researcher has no control over: the result. Further future-proofing of research requires help from expert peers when the study is designed, and not when the dust has already settled.

Enter Registered Reports, a new publication format designed to minimise publication bias and protect research from questionable practices^4^. Registered Reports undergo a two stage peer review process. The first stage of review takes place prior to data collection: editors and reviewers assess the study protocol and help to shape it into the best version of itself. The authors then go away to conduct the study, strictly adhering to the accepted protocol. In the second stage of review, the full paper is evaluated, but the final decision is not dependent on the outcome of the study as long as the protocol was followed and the interpretation of the results is supported by the evidence.

Registered Reports bring a conceptual change in how a lot of experimental research is conducted. The format puts the emphasis on the rigour of methodology and validity of the research question, rather than perceived importance of the findings, and as such encompasses the core values of *Scientific Reports*. We are delighted to now offer Registered Reports at *Scientific Reports* across the full breadth of our scope.

Although the format is often thought of as a solution to problems plaguing hypothesis-driven research, it would be a mistake to think that other fields and types of research cannot benefit from it. All scientists face incentives that can lead to questionable practices; most manuscripts must face cognitive biases of journal editors. Registered Reports don’t just help to deal with these issues; they lead to research that is designed and conducted more robustly, reported more transparently, and communicated without bias. Researchers have very little to lose by adopting Registered Reports. The scientific community has everything to gain.

If you have any questions about your Registered Report submission to *Scientific Reports*, please contact us at scientificreports@nature.com.
